# Chronic Alcohol Drinking Impairs Recognition Memory And Insulin-Associated Genes In The Medial Prefrontal Cortex

**DOI:** 10.1007/s12035-025-05407-1

**Published:** 2025-12-16

**Authors:** Bryan Cruz, Michela Palmisano, Alex Hiroto, Ryan Bullard, Ismael Muñoz Gil, Alexia Anjos-Santos, Angela E. Gonzalez, Celsey M. St. Onge, Valentina Vozella, Roberto Ciccocioppo, Marisa Roberto

**Affiliations:** 1 Department of Translational Medicine, The Scripps Research Institute, La Jolla, CA 92037 USA; 2 San Diego Mesa College, San Diego, CA 92111 USA; 3 Department of Pharmacology, Universidade Federal de São Paulo (UNIFESP), São Paulo, SP Brazil; 4 School of Pharmacy, Center for Neuroscience, Pharmacology Unit, University of Camerino, Camerino, Italy

**Keywords:** Ethanol, Memory, Insulin, Insulin-like growth factor 1, Brain-derived neurotrophic factor, Prelimbic, Infralimbic, Hippocampus CA1

## Abstract

Chronic alcohol drinking increases susceptibility to cognitive impairment; however, the underlying mechanisms remain unclear. In this study, we investigated the effects of chronic alcohol drinking on working and recognition memory in a Marchigian Sardinian alcohol-preferring (msP) rat line. Due to interest in insulin-based medications for alcohol use disorder, we examined insulin/insulin-like growth factor 1 (IGF-1) genes in the prelimbic (PL) and infralimbic (IL) medial prefrontal cortex, a region linked to alcohol dependence and cognition. Male and female msPs received access to alcohol (20% *v/v*) and water (H_2_O) using a group-housed 2 bottle-choice drinking paradigm for several weeks, while controls received H_2_O only. After five weeks, the radial arm maze and novel object recognition tasks evaluated working and recognition memory. At the end of the study, genes encoding for insulin/IGF-1, their receptors, and downstream effectors were assessed in the PL, IL and hippocampus CA1 (CA1), three main regions involved in working and recognition memory processing. Genes regulating brain plasticity were also assessed. Females consumed more alcohol than males. Chronic alcohol exposure selectively impaired recognition memory in males, while working memory remained unaffected in both sexes. Chronic alcohol exposure altered transcription of insulin/IGF-1 signaling components. In females, chronic alcohol reduced *Ins* transcript levels in the IL, while increasing *Insr* expression in the PL, but not in the CA1. In males, chronic alcohol reduced *Igf1r* transcript levels in the IL, but not PL or CA1. Across both sexes and all regions, chronic alcohol decreased *Irs2*, a downstream effector of insulin/IGF-1, transcript levels. Lastly, we observed some alterations in genes linked to memory and plasticity including *Bdnf*, *TrkB*, *Psd95*, and *Pkmζ*. Together, these findings suggest that chronic alcohol drinking impairs recognition memory in males, while broadly disrupting metabolic and plasticity-associated genes in the mPFC and CA1.

**Supplementary Information:**

The online version contains supplementary material available at 10.1007/s12035-025-05407-1.

## Introduction

Alcohol use disorder (AUD) is a major public health concern, linked to a wide range of peripheral and central pathophysiological conditions [1–3]. A growing body of research suggests that AUD negatively impacts cognitive behavior and increases vulnerability to neurodegenerative disorders [4–8]. Despite this evidence, the underlying molecular mechanisms responsible for the long-term effects of AUD on cognitive behavior require more preclinical research. Animal models of AUD continue to provide important screening tools to identify new mechanistic targets and examine molecular adaptions of AUD.

The molecular mechanism of cognitive function broadly encompasses systems involved in neurotrophic signaling and neuronal metabolism as well as neuronal growth and survival [9, 10]. These systems involve cell surface receptors and intracellular mechanisms that include but are not limited to insulin, insulin growth factor-1 (IGF-1), and brain-derived neurotrophic factor (BDNF) [11–13]. Together, these systems converge in PI3k/AKT/mTOR signaling cascades and functionally interconnect to promote phosphorylation of proteins [10, 14–16]. Direct evidence using a rodent model of AUD has found that binge-like alcohol drinking impairs insulin/IGF-1 gene expression in the cerebellum of mice [17]. In other reports, alcohol exposure reduces spatial learning, resulting in decreased receptors for insulin and related downstream effectors in the temporal lobe of rats [18]. In human post-mortem tissue of patients with AUD, insulin gene expression was reduced in the anterior cingulate cortex and vermis portion of the cerebellum versus healthy controls [18]. Furthermore, decreased plasma levels of BDNF and IGF-1 in abstinent patients with AUD have also been observed in clincal settings [19]. However, the mechanistic role of these genetic systems in AUD and cognitive behavior remains unclear and examination of brain regions closely implicated in AUD requires more research.

The medial prefrontal cortex (mPFC), which can be subdivided into the prelimbic (PL) and infralimbic (IL) cortices is essential for higher-order cognitive functions such as decision-making, working memory, and behavioral regulation [20]. The mPFC is an important site for adaptions that occur by AUD [21, 22]. The mPFC has been implicated in cognitive behavior modulated by alcohol dependence. Specifically, alcohol dependent mice displayed impaired retention memory that was accompanied by gene expression changes in the mPFC [23]. Additionally, alcohol dependent rats undergoing abstinence showed impaired cognitive performance on a spatial location discrimination task and increased phosphorylation of calcium/calmodulin-dependent protein kinase in the dorsal, but not ventral mPFC [24]. Further, chronic alcohol drinking impairs mPFC function, in part by activating stress peptide signaling pathways that disrupt associative learning and increase preference to alcohol [22, 25, 26]. There are also distinct differences in PL and IL subdivisions, where the PL is related to expression and consolidation of learned behaviors (e.g. cue-induced drug reinstatement or drug-seeking) and the IL is related to extinction of these learned behaviors [27–30]. Differences in insulin/IGF-1 systems in the mPFC and their possible role in cognitive behavior influenced by chronic alcohol exposure has not been examined.

The present study examined the effects of chronic alcohol drinking on two cognitive domains, working memory and recognition memory, in a genetic model of predisposed high alcohol preference. This well-established strain is known as the Marchigian Sardinian alcohol-preferring (msP) rat line. The msP rats resemble characteristics of AUD and have been genetically mapped over 80 generations for excessive alcohol drinking (> 90% preference and 6–7 g/kg/daily) and stable long-term drinking [31, 32]. This rat line consistently exhibits increased anxiety- and depressive-like behavior, heightened pain sensitivity and stress reactivity compared to their background Wistar strain [32–34]. After drinking and memory testing, we isolated PL and IL tissue and analyzed transcript levels for genes encoding for insulin/IGF-1 and their receptors (*Ins, Insr, Igf1, Igf1r*) as well as their associated downstream intracellular mediators (*Irs1, Irs2*). Furthermore, another important brain region involved in working and recognition memory processing is the hippocampus, which is affected by chronic alcohol exposure [35–37]. Therefore, both insulin/IGF-1 genes and their downstream mediators were also analyzed in hippocampal CA1 (CA1) tissue. To assess common memory- and plasticity- dependent systems, for neurotrophic signaling (*Bdnf, TrkB*) and synaptic potentiation (*Pkmζ, Psd95*) in PL, IL, and the CA1 across all groups. We hypothesized that chronic alcohol exposure would impair learning in both working memory and recognition memory tasks and decrease transcription for insulin/IGF-1 genes in the mPFC and CA1 across both sexes. Further, we hypothesized that the changes produced by chronic alcohol drinking on memory and gene expression would be impaired to a greater degree in females versus males. This hypothesis was based on previous work that females are more sensitive to effects of alcohol and display higher consumption versus males [32].

## Methods

### Rat Strain

Adult male and female msP rats (N = 43) were bred at The Scripps Research Institute (TSRI; La Jolla, CA, USA) and used in this study. The msP colony was previously obtained from the School of Pharmacy, University of Camerino (Camerino, Italy). All rats had *ad libitum* access to food and water. All experimental procedures were approved by TSRI Institutional Animal Care and Use Committee (IACUC; protocol no. 09–0006) and conducted in accordance with the National Institutes of Health Guide for the Care and Use of Laboratory Animals (8th edition).

### Experimental Design & Alcohol Drinking

This study included four groups of adult male and female msP rats that received water (H_2_O; control group) or access to H_2_O and alcohol (alcohol group). The groups were male H_2_O controls (n = 8) or alcohol (n = 11) and female H_2_O control (n = 12) or alcohol (n = 12). Rats in the alcohol groups were first acclimated to alcohol (10% *v/v*) for one week using a two-bottle choice (2BC) paradigm in a group-housed setting (2–3 per cage). In week 2, the alcohol concentration was increased to 20% (*v/v*) and maintained at this concentration for the remainder of the study. Alcohol intake was measured by weighing the bottles every other day (Monday, Wednesday, and Friday starting at 9am) before promptly returning them to the cages. Alcohol intake was expressed by the total alcohol consumption (g) multiplied by the concentration of alcohol (20%) and divided by the total (summation of all rats) body weight (kg) per cage. The age of the rats ranged from post-natal day (PND) 90 to 140 at the time of the initial alcohol exposure. These procedures were adapted from previous studies in genetically selected msP rats, which consistently display high alcohol consumption, averaging approximately 6 g/kg per cage over a 24-h period [32]. Blood alcohol levels were not recorded; however, the expression of high alcohol intake in the msP strain is similar to our original work characterizing this rodent line, with msP rats exhibiting approximately 70–80 mg/dL BALs that peak over 100 mg/dL [32, 38, 39]. The group-housed home cage set-up has been previously used in our laboratory and others [40]. There were 2–3 msP rats per cage.

**Fig. 1:**
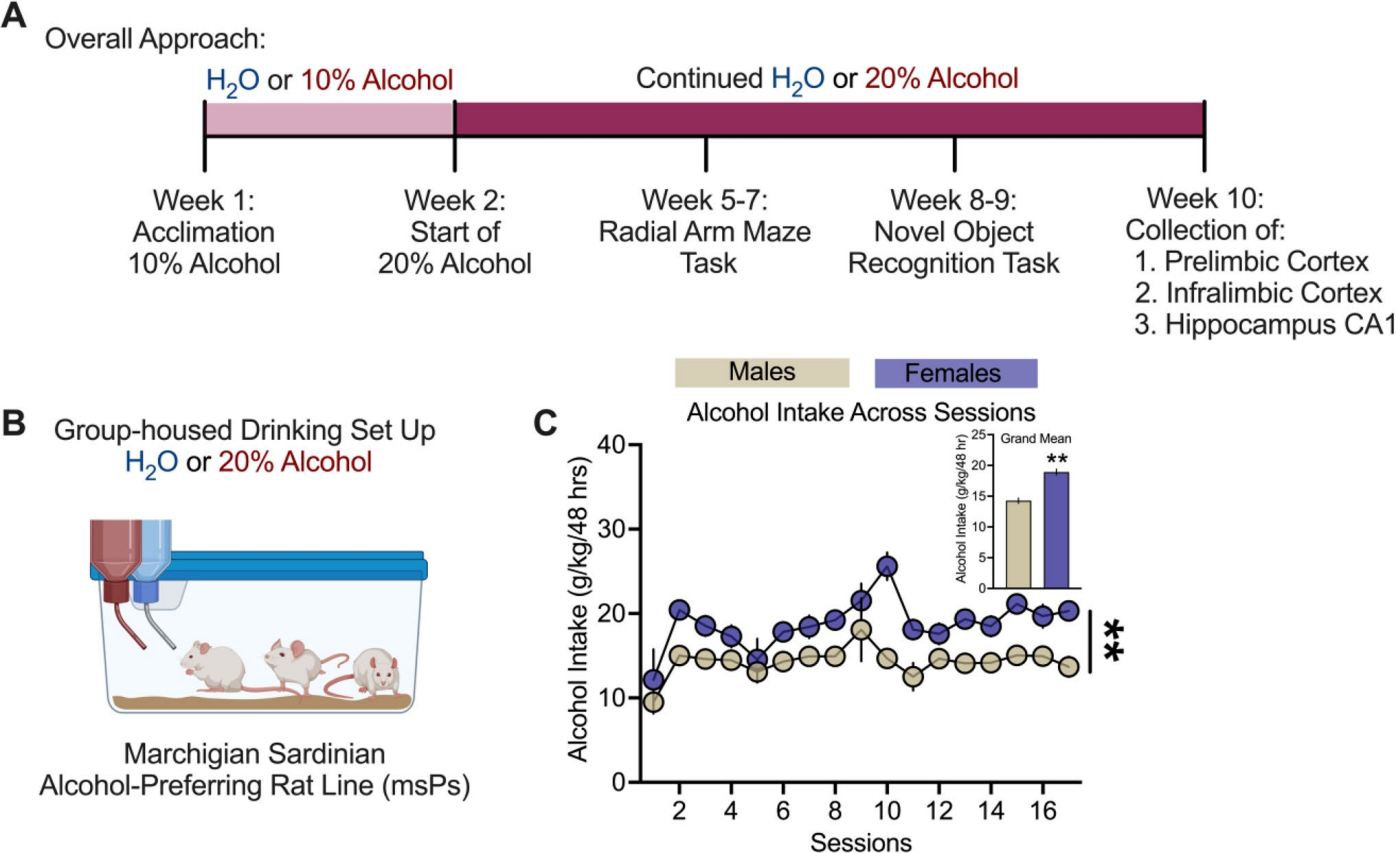
Two-bottle choice drinking in male and female msP rats. **A** Timeline of chronic alcohol drinking regimen, memory testing, and tissue isolation of mPFC subregions, prelimbic (PL) and infralimbic (IL) cortices, and hippocampus CA1 (CA1). **B** Group-housed home cage set-up (2–3 per cage) in msP rats that received *ad libitum* choice of H_2_O alone (controls) and 20% alcohol (v/v) or H_2_O. **C** Alcohol intake (g/kg/48 h) measured over several weeks throughout the drinking regimen and inset of the mean for all sessions. All data were expressed as mean ± SEM and analyzed using repeated measures ANOVA in male (n = 8–11 per group) and female (n = 12 per group) msP groups. The asterisks (*) reflect a significant difference compared to male msP rats, **p < 0.01

From weeks 5 to 7, working memory was assessed using the radial arm maze (RAM) task. Males were tested between weeks 5–6 and females at weeks 6–7. From weeks 8 to 9, recognition memory was evaluated with the novel object recognition (NOR) task. Rats were maintained on either water (H_2_O; control group) or H_2_O and alcohol (alcohol group) *ad libitum* throughout the entire study, including on RAM and NOR test days. Rats were momentarily removed from access to alcohol during their test trial and immediately placed back in their home cage after testing was over. After memory testing, rats remained in their home cages for one additional week with *ad libitum* access to water and alcohol. In week 10, all rats were euthanized, and tissue from the PL, IL, and CA1 were processed for qPCR analysis. See Fig. 1A for the experimental timeline.

### Radial Arm Maze Task

The radial arm maze (RAM) apparatus consisted of eight arms (50 cm long × 10 cm wide × 20 cm wall height) extending from a central platform (30 cm diameter) mounted on a fixed stand. The floors were gray, and the walls were made of clear plexiglass (Maze Engineers®, Skokie, IL). RAM testing included three phases, (1) *acclimation* to chocolate-flavored reward pellets, (2) *habituation* to the maze, with all eight arms baited with reward pellets and (3) *learning* to assess working memory as previously described [41, 42]. In the *acclimation phase*, rats were presented with chocolate-flavored reward pellets in their home cage, while an observer monitored the cage until the pellet was successfully consumed. No other variables were scored. The *habituation phase* served to acclimate rats to the maze where all eight arms were baited with pellets. Rats were allowed to individually explore the maze and consume the pellets in baited arms for 15 min prior to being returned to their home cage. This occurred once on the day prior to the *learning phase*. Time to obtain pellets across any of the baited arms and positioned entries to the baited arms was scored. The *learning phase* consisted of 6 sessions staggered in the morning (starting at 9am) and evening (starting at 3pm) with 2 sessions per day (3 days total). Each session consisted of 3 trials. During each of the trials, only 3 of the 8 arms (randomly assigned for each rat) were baited with positioning consistent across all sessions. Each trial began when the rat was placed on the central platform and ended when the rat retrieved all three rewards or after 3 min had elapsed, which ever came first. Between trials, rats were placed in a holding cage while the maze was cleaned and rebaited. All rats were food-deprived for 4–6 h prior to the habituation and learning phases to improve motivation to seek the pellets during the task. The following dependent variables were recorded, working memory errors (re-entries into previously visited baited arms), reference memory errors (entries into non-baited arms), reward arm entries, total arm entries (baited + non-baited as a measure of general locomotor activity), reward consumption (g), and decision accuracy (ratio of correct baited arm entries to total arm entries). All behavioral data were averaged across the three daily trials and presented as session means in the figures. The chocolate-flavored reward pellets used were 50% sucrose (45 mg, 5TUL, Test Diets, St. Louis, MO, USA).

### Novel Object Recognition Task

The novel object recognition (NOR) task was conducted in a white opaque custom Plexiglas^®^ open-field arena (48 cm long × 48 cm wide × 35 cm wall height). The NOR task was performed over three consecutive days and consisted of the following phases, (1) *habituation*, (2) *familiarization* and (3) *recognition* test. Each phase was separated by a 24 h interval as previously described [43]. On Day 1 (*habituation*), rats were placed individually in the empty open-field arena and allowed to freely explore for 10 min to acclimate to the environment. On Day 2 (*familiarization*)**,** two identical objects (“familiar objects”) were placed in adjacent corners of the arena 5 cm from the intersecting walls. Each rat was placed on the wall opposite the objects, facing away and allowed to explore both the arena and objects for 10 min. On Day 3 (*recognition test*), one of the familiar objects was replaced with a novel object. Rats were placed in the arena in the same manner and allowed to explore for 5 min. Between animals, both the arena and objects were cleaned with 70% ethanol to eliminate olfactory cues that may influence behavior. All NOR testing was conducted starting at 9am. Discrimination Index (DI) was calculated in sec using the formula: $DI=\frac{Time\left(Novel\right)-Time(Familar)}{Time\left(Novel\right)+Time\left(Familiar\right)}$. The DI is an index of recognition memory and lower values indicate memory deficits [44].

### RNA Isolation & qPCR

Rats were anesthetized with isoflurane and rapidly decapitated. Brains were extracted, flash-frozen in dry ice-cooled isopentane, and stored at − 80 °C until further processing. The PL, IL, and CA1 were punched (1 mm diameter) from 400 µm coronal sections. Dissections for the PL began at AP: 3.72 mm; ML: ± 0.45 mm; DV:—3.8 mm, for IL at AP: 3.72 mm; ML: ± 0.45 mm; DV: −5.2 mm, and for CA1 at AP: −3.24 mm; ML: ± 1.2 mm; DV:—2.8 mm from skull and bregma using the Paxinos & Watson Rat Brain Atlas (5th edition). Total RNA was isolated using TRIzol™ reagent (Invitrogen; catalog no. 15596026) followed by purification with RNA extraction kits (Zymo Research; catalog no. NC9972645). Complementary DNA (cDNA) was synthesized using the SuperScript IV VILO™ Master Mix kit with ezDNase (Invitrogen; catalog no. 11766050). Quantitative PCR (qPCR) was performed using PowerTrack™ SYBR Green Master Mix (Applied Biosystems; catalog no. A46109) on a QuantStudio 5 system. Gene expression fold changes were calculated relative to controls using the 2^–∆∆Ct^ method. Data were normalized to either 2 out of the 3 validated housekeeping genes, *Ppia****,**** Actb*, and *Ywhaz* [1]. Two PL and two IL female tissue samples were not included in the assay due to technical error in the RNA isolation. Male and female PCR assays were conducted separately. All primers (forward and reverse) were obtained from Integrated DNA Technologies (Coralville, IA, USA). See Table 1 for primer sequences.

**Table 1:** Forward and reverse primer sequences

Gene	Full name	Forward sequence	Reverse sequence
*Ins*	Insulin	5’-CCGGTGACCTTCAGACCTT-3’	5’-TGCTGGTGCAGCACTGAT-3’
*Insr*	Insulin receptor	5’-TGA CAA TGA GGA ATG TGG GGA-3’	5’-GGG CAA ACT TTC TGA CAA-3’
*Irs1*	Insulin receptor substrate 1	5’-TCACGATTCACAACCAGGAC-3’	5’-AGGGATGCATCGTACCATCT-3’
*Irs2*	Insulin receptor substrate 2	5’-TCACCACAGGACACAGATGC-3’	5’-GCATGAAGTGTGGCAAACGT-3’
*Igf1*	Insulin-like growth factor 1	5’-CAG TTC GTG TGT GGA CCA AG-3’	5′-GTCTTGGGCATGTCAGTGTGG-3′
*Igf1r*	Insulin-like receptor growth factor 1 receptor	5′-TGGCAGAACTGCTGTCTGAG-3′	5′-AACGCAGGGTCTAGTTGAGC-3′
*Bdnf*	Brain-derived neurotrophic factor	5’-GGTCGATTAGGTGGCTTCATAG-3’	5’-CGAACAGAAACAGAGGAGAGATT-3’
*TrkB*	Tyrosine kinase B	5’-CTACCTGGCATCCCAACACT-3’	5’-CTCGGTGGTGAATTTCCTGT-3’
*Pkmζ*	Protein kinase m *ζ*	5′-CCACCTTCGGTAGAGCATAA-3	5′-GCGGTAGATGGACTTGTCTT-3
*Psd95*	Post-synaptic density 95	5′-TAGGGCCCTGTTTGATTACG-3′	5′-TGGCCTTTAACCTTGACCAC-3′
*Actb*	Actin beta	5ʹ-ATCTGGCACCACACCTCC-3ʹ	5ʹ-AGCCAGGTCCAGACGCA-3ʹ
*Ywahz*	tyrosine 3-monooxygenase/tryptophan 5-monooxygenase activation protein zeta	5’-GAAAATGAAGGGTGACTACTAC-3’	5’-CTGATTTCAAATGCTTCTTGG-3’
*Ppia*	Peptidylprolyl isomerase A	5’-TTTGGGAAGGTGAAAGAAGGC-3’	5’-ACAGAAGGAATGGTTTGATGGG-3’

### Statistics

Alcohol consumption data were analyzed using a two-way repeated measures analysis of variance (ANOVA) with Sex (Male vs. Female) as the between-subjects factor and Session (Days 1–17) as the within-subjects factor. RAM data were analyzed using a two-way repeated measures ANOVA with Group (H_2_O vs. Alcohol; labeled as “Alcohol” in Tables S1 and and S2) as the between-subjects factor and Learning Session (1–6) as the within-subjects factor. NOR and gene expression data were analyzed using independent samples *t*-tests, conducted separately for each sex. All data was tested for assumptions of normality using the Shapiro–Wilk test. In instances, where assumptions of normality were not met, the non-parametric Mann–Whitney U test was used and reflected in the text. All statistical values including test statistics, degrees of freedom, and *p*-values are indicated in Table S1 of the supplementary, thus, only *p*-values are reported throughout the main text. A separate three-way or two-way ANOVA was conducted as appropriate to examine potential sex differences across alcohol groups on RAM and NOR outcomes in Table S2.

All results are reported as mean ± SEM. Statistical significance was set at *p* < 0.05 with a 95% confidence interval. Significant outliers as defined by a *p* < 0.05 in the Grubbs’ test were removed from the analysis. Analyses and graphing were performed using GraphPad Prism (version 10.3.1) and IBM SPSS Statistics (version 30). All experimenters were blind to the group conditions in the behavioral testing and qPCR assay.

## Results

### Two Bottle Choice Drinking in Male and Female msP Rats

We used a two-bottle choice paradigm to assess whether chronic alcohol drinking negatively impacts working and recognition memory. Our procedural timeline and drinking set**-**up are indicated in Fig. 1 A, B. Our results revealed that *ad libitum* access to alcohol (20% *v/v*) consumption (g/kg/48 h) was significantly higher in females as compared to males across 17 recorded sessions (Fig. 1 C, *p* = 0.003).

### Effects of Chronic Alcohol Drinking On Radial Arm Maze Performance in Male and Female msP Rats

**Fig. 2:**
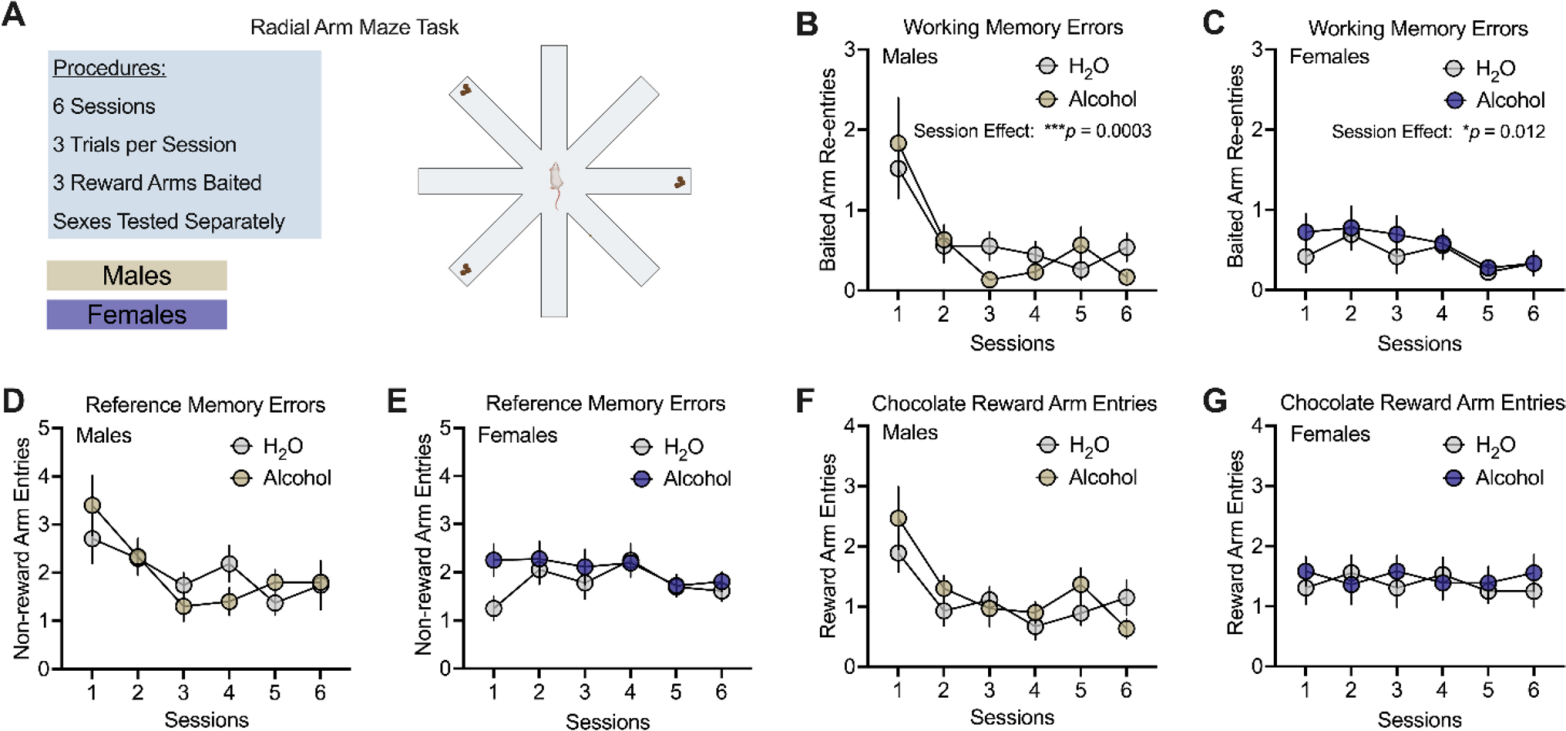
Effects of chronic alcohol drinking on radial arm maze performance in male and female msP rats. **A** Procedures and apparatus of the radial arm maze (RAM) task. In males and females: **B, C** reflect working memory errors; **D, E** reflect reference memory errors; **F, G** reflect reward arm entries. All data were averaged across the 3 trials per each session, expressed as mean ± SEM and analyzed using two-way repeated measures ANOVA across male (n = 8–11 per group) and female (n = 12 per group) msP groups. The asterisks (*) reflect a significant difference across session, regardless of alcohol exposure, *p < 0.05 or ***p < 0.001

Fig. 2 A shows experimental procedures and apparatus for the RAM task in alcohol exposed as well as control male and female msP rats. There was a main effect of session with fewer working memory errors made over time in all groups (Fig. 2 B, C, *p’s* < 0.05). Specifically, both alcohol and H_2_O control groups displayed normal learning performance and showed no difference in working memory errors (Fig. 2 B, C, *p’s* > 0.05), reference memory errors (Fig. 2 D, E, *p’s* > 0.05) or reward arm entries (Fig. 2 F, G, *p’s* > 0.05)

Other general task performance measures were scored including decision accuracy (%), reward pellets consumed (g), and total arm entries. No differences were observed between alcohol and control groups across sex for decision accuracy (Fig. S1 A, D, *p’s* > 0.05), total rewards consumed (Fig. S1 B, E, *p’s* > 0.05), and total arm entries (Fig. S1 C, F, *p’s* > 0.05). A separate analysis was conducted to examine possible sex differences across alcohol groups on RAM outcomes in Table S2. In general, our analysis revealed that there were no differences by sex and alcohol in msPs across repeated sessions assessments (1–6) on working memory errors, reference memory errors, chocolate reward arm entries, decision accuracy, rewards consumed, or total arm entries (Table S2, *p's* > 0.05).

### Effects of Chronic Alcohol Drinking On the Novel Object Recognition Task in Male and Female msP Rats

**Fig. 3:**
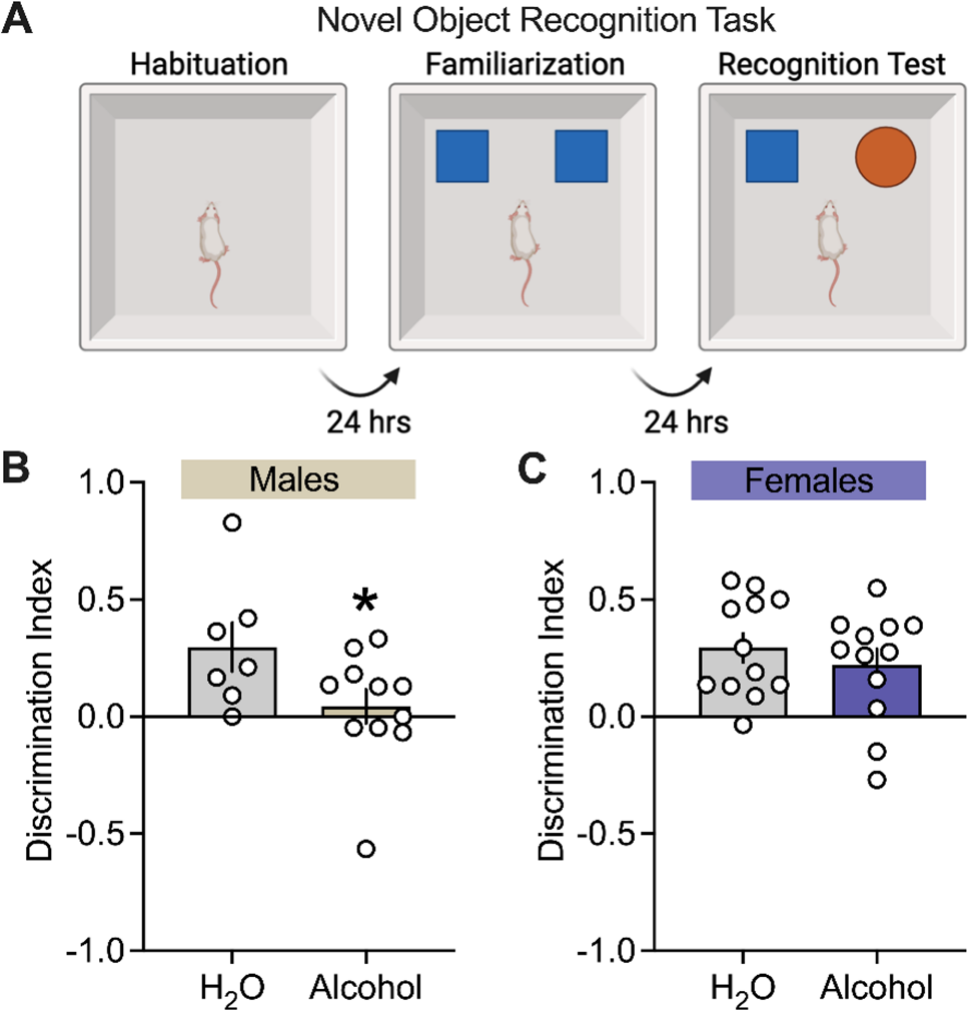
Effects of chronic alcohol drinking on the novel object recognition task in male and female msP rats. **A** Procedure and apparatus of the novel object recognition (NOR) task. In males and females: **B, C** reflect discrimination index (DI) score. DI score was calculated by comparing the amount of time exploring the novel object versus the familiar object. All data were expressed as mean ± SEM and analyzed using *t*-test or non-parametric Mann–Whitney U independently in male (n = 7–11 per group) and female (n = 12 per group) msP groups. The asterisks (*) reflect a significant difference between alcohol groups and their respective H_2_O controls, * p < 0.05

Figure 3A shows the experimental procedures used for the NOR task performed in alcohol-exposed as well as H_2_O control male and female msP rats. Recognition memory was quantified using a discrimination index (DI). Chronic alcohol exposure resulted in a sex-dependent effect on DI. Specifically, male rats with a history of alcohol exposure had a significantly lower DI compared to H_2_O controls (Fig. 3 B, *p* = 0.029). In contrast, there was no difference in DI between alcohol and control groups in females (Fig. 3** C**, *p* = 0.22). There was also no difference in DI between sex and alcohol (Table S2, *p's* > 0.05).

### Effects of Chronic Alcohol Drinking On Insulin-related Genes in the mPFC and Hippocampus CA1 Regions of Male and Female msP Rats

**Fig. 4:**
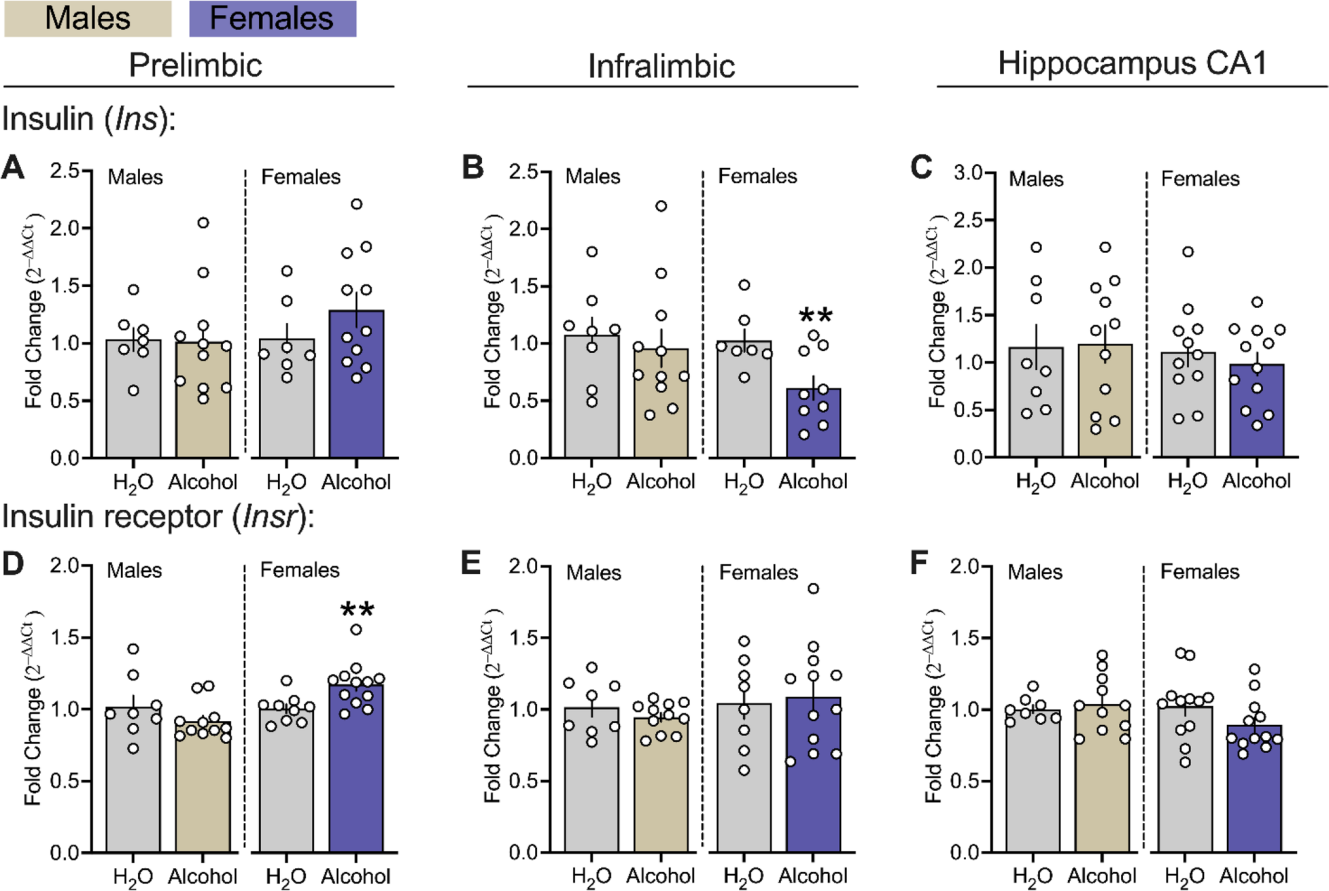
Effects of chronic alcohol drinking on insulin-related genes in the mPFC and hippocampal CA1 of male and female msP rats.** A, B** and **C** reflects gene encoding for insulin (*Ins*). **D, E** and** F** reflects gene encoding for insulin receptor (*Insr*). All data were expressed as fold change relative to controls, transformed using the 2^–∆∆Ct^ method, and analyzed using *t*-tests or Mann Whitney U tests, as appropriate, independently in male (n = 7–11 per group) and female (n = 7–12 per group) msP groups. All PCR assays were conducted separately for each sex. The asterisks (*) reflect a significant difference between alcohol groups and their respective H_2_O controls, ** p < 0.01

Figure 4 shows the effects of chronic alcohol exposure on insulin-related genes, insulin (*Ins*) and insulin receptor (*Insr*), within the PL and IL subregions of the mPFC as well as the CA1 in male and female msP rats. Chronic alcohol drinking decreased IL *Ins* and increased PL *Insr* transcript levels in female alcohol-exposed msPs versus H_2_O controls (Fig. 4 B right panel, females: *p* = 0.006; Fig. 4 D right panel, females: *p* = 0.005). No changes were observed in PL *Ins*, IL *Insr* or in CA1 region in females or any of these genes in male groups (Fig. 4 A-F, *p’s* > 0.05).

### Effects Of Chronic Alcohol Drinking On Insulin-like Growth Factor 1 in the mPFC and Hippocampus CA1 Regions of Male and Female msP Rats.

**Fig. 5:**
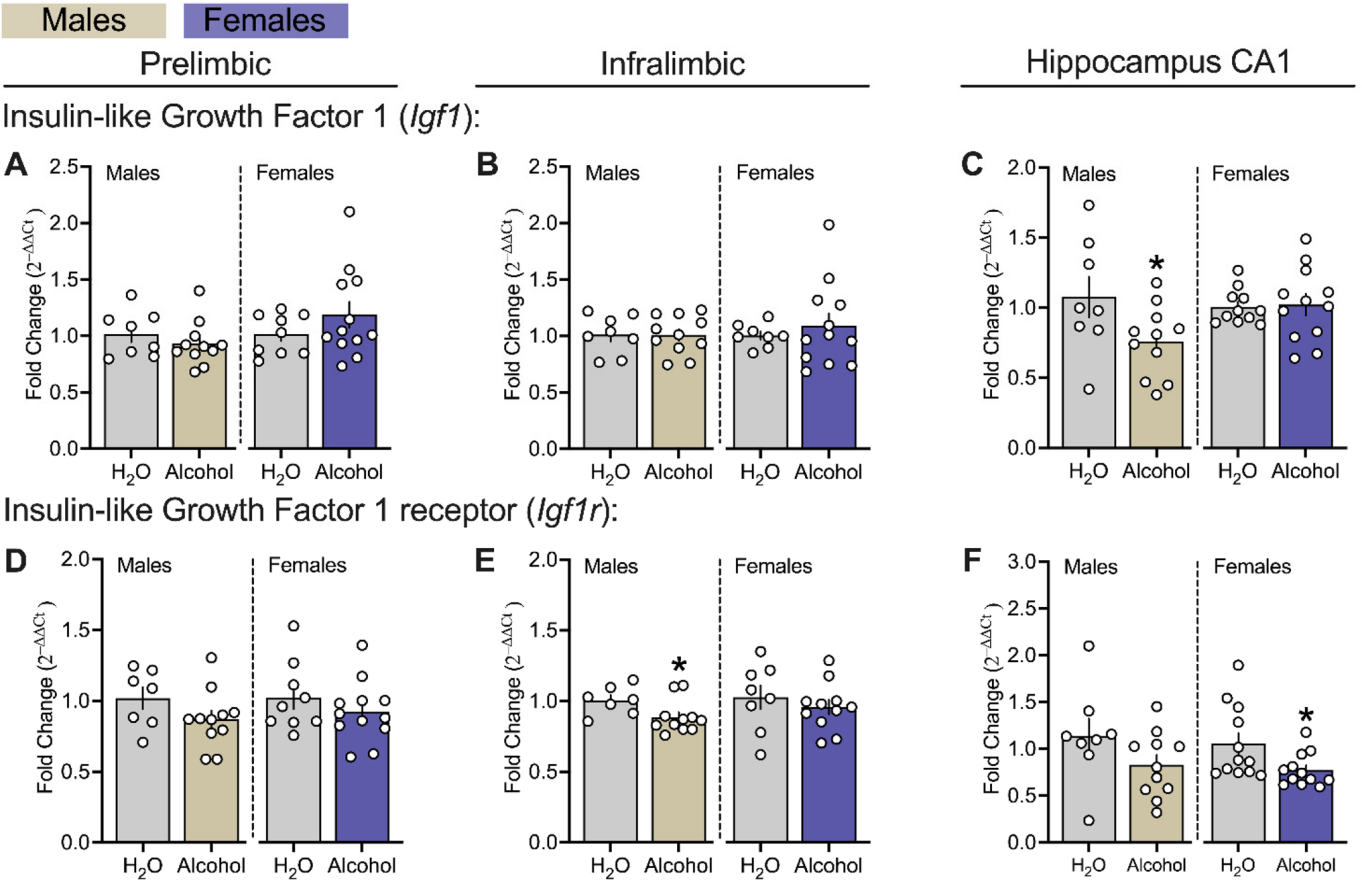
Effects of chronic alcohol drinking on insulin-like growth factor 1 genes in the mPFC and hippocampus CA1 of male and female msP rats.** A, B** and **C** reflects gene encoding for insulin-like growth factor 1(*Igf1*). **D, E** and** F** reflects gene encoding for insulin-like growth factor 1 receptor (*Igf1r*). All data were expressed as fold change relative to controls, transformed using the 2^–∆∆Ct^ method, and analyzed using *t*-tests or non-parametric Mann–Whitney U test independently in male (n = 7–11 per group) and female (n = 8–12 per group) msP groups. All PCR assays were conducted separately for each sex. The asterisks (*) reflect a significant difference from their respective H_2_O controls, *p < 0.05

Figure 5 displays the effects of chronic alcohol exposure on genes associated with insulin-like growth factor 1 (IGF-1; *Igf1*) and IGF-1 receptor (*Igf1r*), within the PL and IL subregions of the mPFC as well as the CA1 in male and female msP rats. Chronic alcohol drinking selectively decreased IL *Igf1r* transcript levels in male alcohol-exposed msPs versus H_2_O controls (Fig. 5 E left panel, males: *p* = 0.021). This effect on *Igf1r* was not observed in PL and CA1 of msP males (Fig. 5 D left panel, males: *p* > 0.05; Fig. 5 F left panel, males: *p* > 0.05) or the PL and IL of female msPs (Fig. 5 D, E right panels, *p*’s > 0.05). Within the CA1, chronic alcohol drinking decreased *Igf1* transcript levels in males and *Igf1r* in females as compared to their respective H_2_O controls, (Fig. 5 C left panel, males: *p* = 0.026; Fig. 5 F right panel, females: *p* = 0.012). Lastly, our analysis did not reveal changes in *Igf1* transcript levels in subregions of the mPFC (Fig. 5 A, B, *p*’s > 0.05) or in the CA1 of females (Fig. 5 C right panel, *p* > 0.05).

### Effects of Chronic Alcohol Drinking On Downstream Effectors of Insulin and Insulin-like Growth Factor 1 in the mPFC and Hippocampus CA1 Regions of Male and Female msP Rats.

**Fig. 6:**
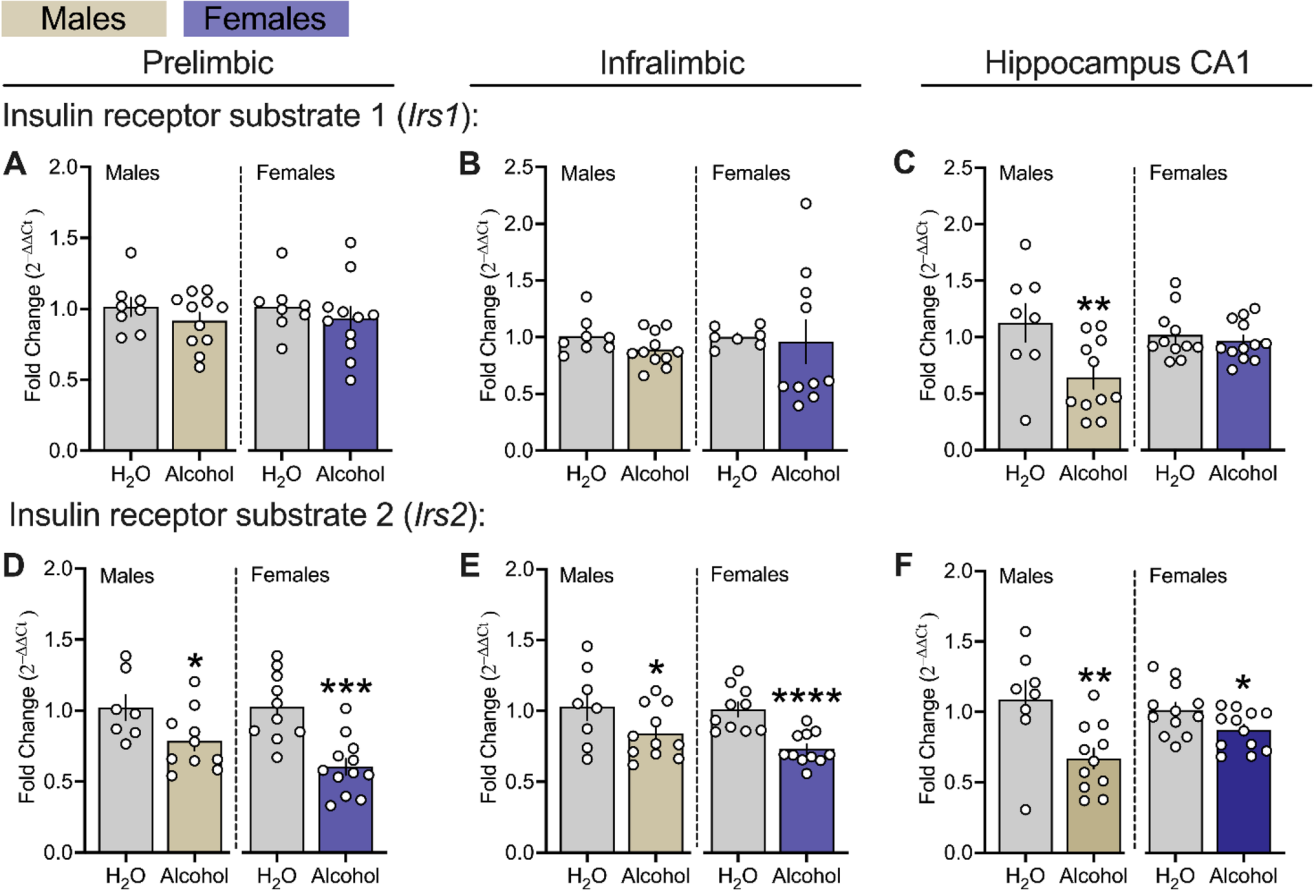
Effects of chronic alcohol drinking on downstream effectors of insulin and insulin-like growth factor 1 in the mPFC and hippocampus CA1 of male and female msP rats. **A, B** and** C** reflects gene encoding for** i**nsulin receptor substrate 1 (*Irs1*). **D, E** and** F** reflects gene encoding for insulin receptor substrate 2 (*Irs2*). All data were expressed as fold change relative to controls, transformed using the 2^–∆∆Ct^ method, and analyzed using *t*-tests independently in male (n = 7–11 per group) and female (n = 7–12 per group) msP groups. All PCR assays were conducted separately for each sex. The asterisks (*) reflect a significant difference from their respective H_2_O controls, *p < 0.05, ***p < 0.001, or ****p < 0.0001

Figure 6 displays the effects of chronic alcohol exposure on downstream insulin/IGF-1 signaling, via transcript levels of insulin receptor substrate 1 (*Irs1*) and insulin receptor substrate 2 (*Irs2*), within the PL and IL subregions of the mPFC and CA1 in male and female msP rats. Our analysis revealed that chronic alcohol drinking decreased PL, IL and CA1 *Irs2* transcript levels in both male and female msPs when compared to their respective controls (Fig. 6 D left panel, males: *p* = 0.024 and right panel, females: *p* = 0.0001; Fig. 6 E left panel, males: *p* = 0.041 and right panel, females *p* = 0.0001; Fig. 6 F left panel, males: *p* = 0.004 and right panel, females: *p* = 0.019). There was no effect of alcohol on PL or IL *Irs1* transcript levels in either male or female msP groups (Fig. 6 A, B both panels, *p’s* > 0.05), however, there was a reduction of this gene in the CA1 of males but not females (Fig. 6 C left panel, males: *p* = 0.008, and right panel, females: *p’s* > 0.05).

### Effects of Chronic Alcohol Drinking On Neurotrophic Signaling Genes in the mPFC and Hippocampus CA1 Regions of Male and Female msP Rats

**Fig. 7:**
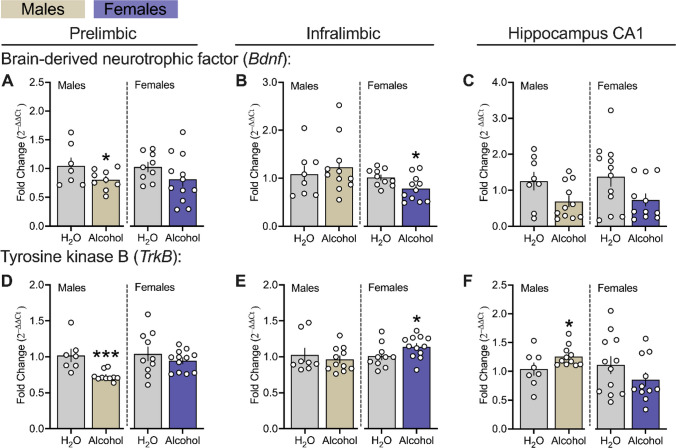
Effects of chronic alcohol drinking on neurotrophic signaling genes in the mPFC and hippocampus CA1 of male and female msP rats. **A, B** and **C** reflects gene encoding for brain derived neurotrophic factor (*Bdnf*). **D, E** and** F** reflects gene encoding for tyrosine kinase B (*TrkB*). All data were expressed as fold change relative to controls, transformed using the 2^–∆∆Ct^ method, and analyzed using *t*-tests or non-parametric Mann–Whitney U independently in male (n = 7–11 per group) and female (n = 9–12 per group) msP groups. All PCR assays were conducted separately for each sex. The asterisks (*) reflect a significant difference from their respective H_2_O controls, *p < 0.05 or ***p < 0.001

In Fig. 7**,** we examined genes encoding for brain-derived neurotrophic factor (*Bdnf)* and its receptor, tyrosine kinase B (*TrkB*) within the PL and IL subregions of the mPFC and CA1 in male and female msP rats*.* Chronic alcohol drinking decreased PL *Bdnf* transcript levels in males, while decreasing IL *Bdnf* in females as compared to H_2_O controls (Fig. 7 A left panel, males: *p* = 0.048; Fig. 7 B right panel, females: *p* = 0.016). Additionally, PL *TrkB* transcript levels were decreased in alcohol-exposed males, while IL *TrkB* transcript levels were increased in alcohol-exposed females as compared to H_2_O controls (Fig. 7 D left panel, males: *p* = 0.0002; Fig. 7 E right panel, females: *p* = 0.037). In the hippocampus CA1, chronic alcohol drinking increased *TrkB* transcript levels in males as compared to H2O controls (Fig. 7 F right panel, males: *p* = 0.043). There were no differences observed in PL *TrkB* of females or IL *Bdnf* and *TrkB* of males after chronic alcohol exposure (Fig. 7 D right panel and E left panel, *p’s* > 0.05). Lastly, there were also no differences observed in CA1 *Bdnf* or in *TrkB* levels in females (Fig. 7 C both panels and F right panel, *p’s* > 0.05).

### Effects of Chronic Alcohol Drinking On Synaptic Potentiation-related Genes in the mPFC and Hippocampus CA1 Regions of Male and Female msP Rats

**Fig. 8:**
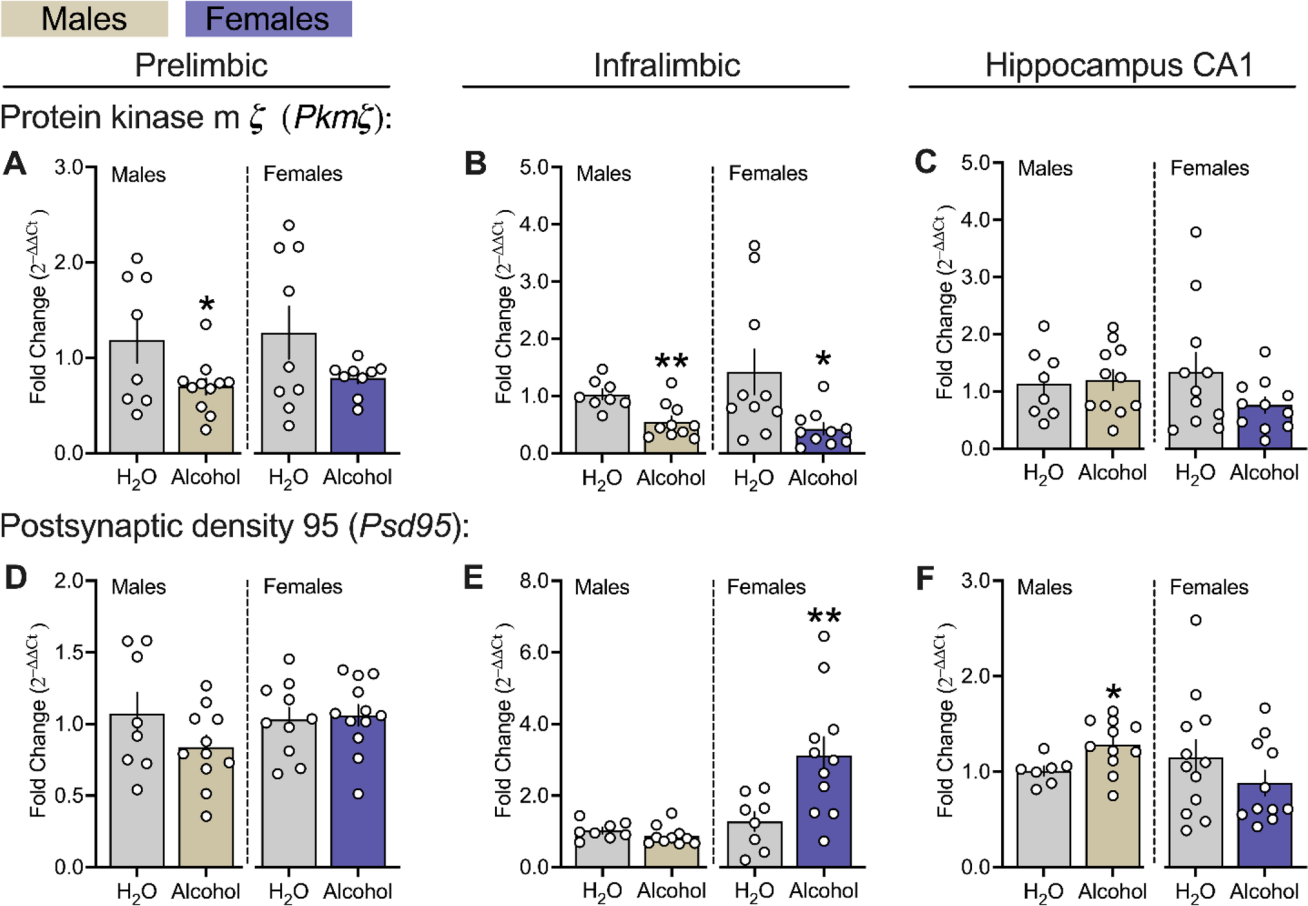
Effects of chronic alcohol drinking on synaptic potentiation-related genes in the mPFC and hippocampus CA1 of male and female msP rats.** A, B** and** C** reflects gene encoding for protein kinase m ζ (*Pkmζ*). **D, E** and** F** reflects gene encoding for postsynaptic density 95 (*Psd95*). All data were expressed as fold change relative to controls, transformed using the 2^–∆∆Ct^ method, and analyzed using *t*-tests or non-parametric Mann–Whitney U independently in male (n = 8–11 per group) and female (n = 9–12 per group) msP groups. All PCR assays were conducted separately for each sex. The asterisks (*) reflect a significant difference from their respective H_2_O controls, *p < 0.05 or **p < 0.01

Figure 8 displays the effects of chronic alcohol exposure on synaptic potentiation encoding genes for protein kinase m ζ (*Pkmζ*)*,* and postsynaptic density 95 (*Psd95*), within the PL and IL subregions of the mPFC, as well as the CA1 in male and female msP rats. Chronic alcohol drinking decreased PL *Pkmζ* transcript levels in alcohol-exposed male msPs, while also decreasing IL *Pkmζ* alcohol exposed male and female msPs as compared to H_2_O controls(Fig. 8 A left panel, males: *p* = 0.022; Fig. 8 B, left panel males: *p* = 0.001 and right panel females: *p* = 0.007). In addition, chronic alcohol drinking increased IL *Psd95* transcript levels in females versus H_2_O controls (Fig. 8 E right panel, females: *p* = 0.009), but had no effect on males in the IL or across either sex in the PL (Fig. 8 D both panels, *p* ‘s > 0.05; Fig. 8 E left panel, males: *p* > 0.05). In the CA1, exposure to chronic alcohol increased IL *Psd95* in males as compared to H_2_O controls, but not in female groups (Fig. 8 F left panel, males: *p* = 0.012, right panel, females: *p* > 0.05). There were no differences in hippocampal CA1 for *Pkmζ* across both male and female msP groups (Fig. 8 C both panels, male and females: *p*’s > 0.05).

## Discussion

The present study investigated the effects of chronic alcohol drinking on two memory domains, working and recognition memory, across male and female msP rats. After behavioral testing, we examined genes encoding for insulin, IGF-1, and BDNF systems in the PL and IL subdivisions of the mPFC as well as the hippocampus CA1. Our results showed that females consumed more alcohol than males in 2BC procedures as previously reported [32]. Chronic alcohol drinking did not impair working memory in either sex, however, it selectively impaired recognition memory in males. Our gene expression analysis in females revealed that chronic alcohol reduced IL *Ins* transcript levels, while increasing PL *Insr* expression, with no changes observed in the CA1 region. Chronic alcohol reduced IL *Igf1r* and CA1 *Igf1* transcript levels in males as well as CA1 *Igf1r* transcript levels in females. Across all sexes and regions, chronic alcohol decreased the expression of *Irs2*, a downstream effector of the insulin/IGF-1 systems. Regarding BDNF, chronic alcohol drinking decreased PL *Bdnf* and *TrkB* in males, while in females, IL *Bdnf* was decreased and *TrkB* was increased. Additionally, chronic alcohol increased CA1 *TrkB* in males. Lastly, our analysis of *Psd95* and *Pkmζ* of these genes across PL, IL, and CA1 regions of some groups after chronic alcohol drinking, which suggest that the long-term effects of alcohol broadly impact other structures.

Our hypothesis was that chronic alcohol exposure would impair both working and recognition memory in male and female msP rats, and to a greater degree in females. However, our results revealed impaired recognition memory in males, while females remained unaffected. Further, no significant differences were observed in working memory performance for either sex. In line with our results, some studies have shown that chronic alcohol impairs long-term memory but not short-term memory in adolescent mice, or that acute alcohol exposure disrupts memory acquisition but not consolidation [45–47]. Additionally, one study reported that acute alcohol impairs object recognition memory in males only, while females appeared resistant to these effects [48]. These findings suggest that the impact of alcohol on memory function may be mediated by multiple interacting factors such as age, rodent or genetic strain, and the magnitude of alcohol intake which together may influence the extent of memory disruption. Taken together, these results point to the importance of considering the aforementioned variables when performing alcohol-related memory studies.

Although females consumed greater amounts of alcohol compared to males, females appeared resilient to alcohol-induced memory impairment**,** particularly in the recognition memory task. Emerging evidence indicates that female memory performance may be modulated by additional biological factors such as hormonal status [49]. In one study, examination of naturally cycling alcohol-exposed females demonstrated poorer performance in the proestrus phase compared to other phases on the object recognition task [48]. However, the same study reported that no differences in recognition memory by acute alcohol exposure when all phases were considered together [48]. Although we did not assess the estrous phase, it is speculated that hormonal fluctuations in females may have interacted with or masked memory deficits. In support of this, prior studies have found that female rats perform better on recognition tasks during metestrus and proestrus, while alcohol-induced memory impairments are most evident during proestrus, but not during estrus [48, 50]. Importantly, estrogen has also been shown to enhance memory function, specifically in hippocampal mechanisms [51]. These findings highlight the importance of accounting for hormonal cycles in alcohol-memory research and future studies should incorporate estrous cycle monitoring to more accurately assess sex-specific vulnerability to alcohol-related memory impairment.

We examined key genes involved in insulin/IGF-1 signaling, which play critical roles in neural metabolism and are disrupted by chronic alcohol exposure [18, 52–54]. The most striking difference was that chronic alcohol disrupted transcript levels for *Ins* in the IL (decreased) and its receptor, *Insr* in the PL (increased), in females but not males. No differences were observed in *Ins* or *Insr* in the CA1 of msPs. In contrast, chronic alcohol exposure decreased transcript levels for *Igf1r* in the IL of males and in the CA1 of females, but not in the PL or IL female groups. These findings suggest a possible separation of molecular adaptions of subregions of the mPFC and CA1. Although the central mechanisms linking insulin/IGF-1 signaling to alcohol dependence remain unclear, we speculate that the long-term consequence of alcohol consumption suppresses these systems which may contribute to changes in cognitive behavior. As such, one report in alcohol dependent patients found lower serum insulin levels that correlated with slower cognitive functions [55]. It has also been shown that intranasal insulin improved memory and executive function in clinical settings [56, 57]. Future studies are needed to investigate the application of insulin/IGF-1 as it may normalize alcohol-related memory impairments, particularly in males. We acknowledge that insulin and IGF-1 are separate systems with low affinity for binding to their respective receptors, however, the convergence of shared downstream targets is critical as a possible sight of action for either sex.

The expression of intracellular downstream mediators of both insulin and IGF-1 signaling, *Irs1* and *Irs2,* were also tested in the PL, IL, and CA1 following chronic alcohol exposure in msP rats. We observed a consistent and robust reduction in *Irs2* transcript levels across all regions and sexes, suggesting that *Irs2* may be particularly sensitive to the effects of chronic alcohol exposure and possibly alter recognition memory in males. We also observed reductions of *Irs1* transcript levels only in alcohol-exposed male msP rats. This persistent downregulation of *Irs2* is especially intriguing, as it may reflect a broader impairment of downstream insulin/IGF-1 signaling, potentially indicating alcohol-induced desensitization to insulin or IGF-1 at the cellular level. Although the role of *Irs2* on cognitive behavior within the context of alcohol exposure has not been tested, its involvement in learning and memory has been demonstrated in other models. Whole-brain deletion of *Irs2* enhanced memory formation in a spatial learning task [58]. Conditioning studies overexpressing *Irs2* enhanced cue-related drug-seeking for cocaine, while silencing *Irs2* led to aversion or reduced preference for opiates, particularly in the ventral tegmental area (VTA) [59–61]. These studies suggest a dynamic involvement of *Irs2* which may be differentially affected across drug (stimulant versus sedative) and knock-out models in the presence or absence of these drugs.

**Fig. 9:**
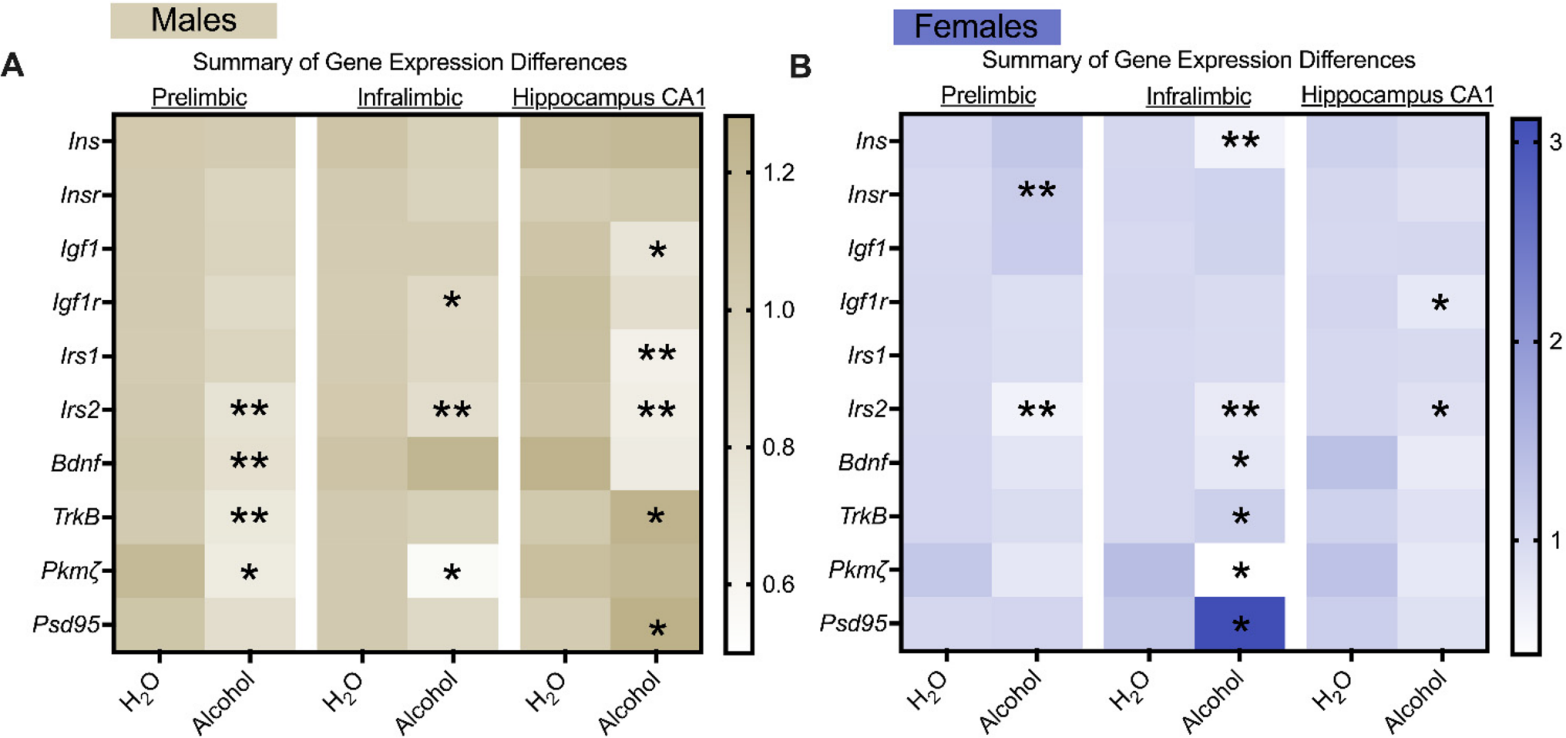
Summary of gene expression changes distributed in a heat map in the mPFC and hippocampus CA1 of male and female msP rats.** A** and** B** Heat map across all genes analyzed in male and female msPs. Mean changes on the fold change relative to controls and transformed using the 2^–∆∆Ct^ method was used for the heat map. The same analysis is reflected in male (n = 8–11 per group) and female (n = 9–12 per group) msP groups. All PCR assays were conducted separately for each sex. The asterisks (*) reflect a significant difference from their respective H_2_O controls, *p < 0.05, **p < 0.01, or ***p < 0.0001

To assess memory dependent systems, we analyzed genes encoding for synaptic potentiation (*Pkmζ* and *Psd95*) and neurotrophic signaling (*Bdnf, TrkB*) across PL, IL and CA1 tissue. We found that chronic alcohol drinking decreased *Pkmζ* transcript levels in the PL and IL of males and in the IL of females. *Psd95* levels were elevated in the IL of alcohol-exposed females and CA1 of males. It is challenging to reconcile these gene changes with our behavioral findings since they occurred in both sexes while recognition memory was only affected in alcohol-exposed males. It was also observed that changes in *Bdnf* and *TrkB* levels in alcohol-exposed msPs were stronger in the PL of males and IL of females, an effect that was marginal in the CA1. However, one important observation is that across all genes analyzed, alcohol-exposed males exhibited greater differences in the CA1 (see Fig. 9). These findings suggest broad impairment of these systems by chronic alcohol consumption in memory-dependent structures and might explain differences observed in recognition memory, specifically in males. Similar to the PL and IL for *Irs2*, chronic alcohol drinking also decreased CA1 *Irs2* levels in both male and female msPs, further highlighting the possible role of this gene in alcohol-related cognitive behavior.

There are some limitations to consider from this study. First, our design integrated a group-housed drinking model over individually housed rats. This approach does not allow for analysis of individual relationships between our behavioral or gene outcomes and alcohol drinking levels. Thus, we are unable to rule out how variations in sex or alcohol consumption may predict cognitive performance or gene expression changes. Second, we acknowledge that chronic alcohol drinking may have a broader impact in brain structures beyond the mPFC and CA1, which may influence the behavioral results in this study. Third, we did not assess how chronic alcohol drinking may impact the background Wistar strain, which are typically considered lower drinking controls. However, the goal of the study was to assess the negative impact of chronic alcohol exposure in a rodent model that translationally mimics AUD, which is not captured in the Wistar rats using standard non-dependent 2BC procedures. Furthermore, there was no functional assessment on how these gene results may modulate alcohol-related memory impairment. Future studies will test the direct role of insulin/IGF-1 systems in the mPFC or CA1 to modulate the effects of chronic alcohol drinking and cognitive behavior.

In summary, this study demonstrated that chronic alcohol consumption impairs recognition memory in males, but not females**.** We observed persistent downregulation of insulin/IGF-1 gene expression largely due to chronic alcohol exposure. Assessment of memory dependent systems found alterations in *Pkmζ* and *Psd95* gene expression distinctly in subregions of mPFC and CA1 in groups of alcohol exposed-msPs. This study primarily focused on mPFC as a substrate for alcohol-related cognitive behavior. The rationale to examine the mPFC is based on emerging reports studying alcohol dependent effects on working memory and mPFC gene expression [23, 62, 63]. We consider that the hippocampus also contributes to changes associated with alcohol and recognition memory [64–66]. Targeting metabolic- and plasticity- related systems by augmenting insulin and IGF-1 signaling may be considered a therapeutic strategy for reducing AUD-related cognitive deficits. In recent years, medications that indirectly control secretion of insulin (e.g. glucagon-like peptide 1 receptor agonist [GLP-1r]) have gained interest as a potential therapeutic intervention for AUD, prompting our laboratory to investigate insulin/IGF-1 systems for its potential role in alcohol-related memory disruptions [67–69]. One report found that Semaglutide, a GLP-1r agonist, improved cognitive behavior in a genetic mouse model of Alzheimer disease [70]. A recent clinical trial has proposed the application of intranasal insulin for AUD [71]. This report also hypothesized a framework for using insulin to restore cognitive and executive function in patients with AUD, highlighting the possible role of the targets assessed in this study.

## Supplementary Information

Below is the link to the electronic supplementary material.

Supplementary file1 (DOCX 1744 KB)

## Data Availability

No datasets were generated or analysed during the current study.
