# Supplementary material for: Chronic Alcohol Drinking Impairs Recognition Memory And Insulin-Associated Genes In The Medial Prefrontal Cortex
Source: Mol Neurobiol. 2025 Dec 16;63(1):300. doi: 10.1007/s12035-025-05407-1 (PMC12708821; doi:10.1007/s12035-025-05407-1)
Supplement: Supplementary file 1 — Supplementary file1 (DOCX 1744 KB) [file 12035_2025_5407_MOESM1_ESM.docx]

**Supplementary Results:**

**Title:** Chronic alcohol drinking impairs recognition memory and insulin-associated genes in the medial prefrontal cortex

**Authors:** Bryan Cruz^1‡^, Michela Palmisano^1^, Alex Hiroto^1^, Ryan Bullard^1^, Ismael Muñoz Gil^2^, Alexia Anjos-Santos^1,3^, Angela E. Gonzalez^1^, Celsey M. St. Onge^1^, Valentina Vozella^1^, Roberto Ciccocioppo^3^, & Marisa Roberto^1‡^

**Affiliation:** ^1^Department of Translational Medicine, The Scripps Research Institute, La Jolla, CA, 92073, USA; ^2^San Diego Mesa College, San Diego, 92111, USA; ^3^Department of Pharmacology, Universidade Federal de São Paulo (UNIFESP), São Paulo, SP, Brazil; ^4^School of Pharmacy, Center for Neuroscience, Pharmacology Unit, University of Camerino, Camerino, Italy.

**Joint Correspondence:** ^‡^Bryan Cruz, Ph.D., Department of Translational Medicine, The Scripps Research Institute; 10550 N. Torrey Pines Road, La Jolla, CA 92037, Email: [bcruz@scripps.edu](mailto:bcruz@scripps.edu); ^‡^Marisa Roberto, Ph.D., Department of Translational Medicine, The Scripps Research Institute; 10550 N. Torrey Pines Road, La Jolla, CA 92037, Email: [mroberto@scripps.edu](mailto:mroberto@scripps.edu)

**Acknowledgments:** The authors thank Mrs. Jessica Benedict for her technical assistance and Dr. Amanda J. Roberts for her input on memory testing.

**Keywords:** ethanol, memory, insulin, insulin-like growth factor 1, brain derived neurotrophic factor, prelimbic, infralimbic, hippocampus CA1

**
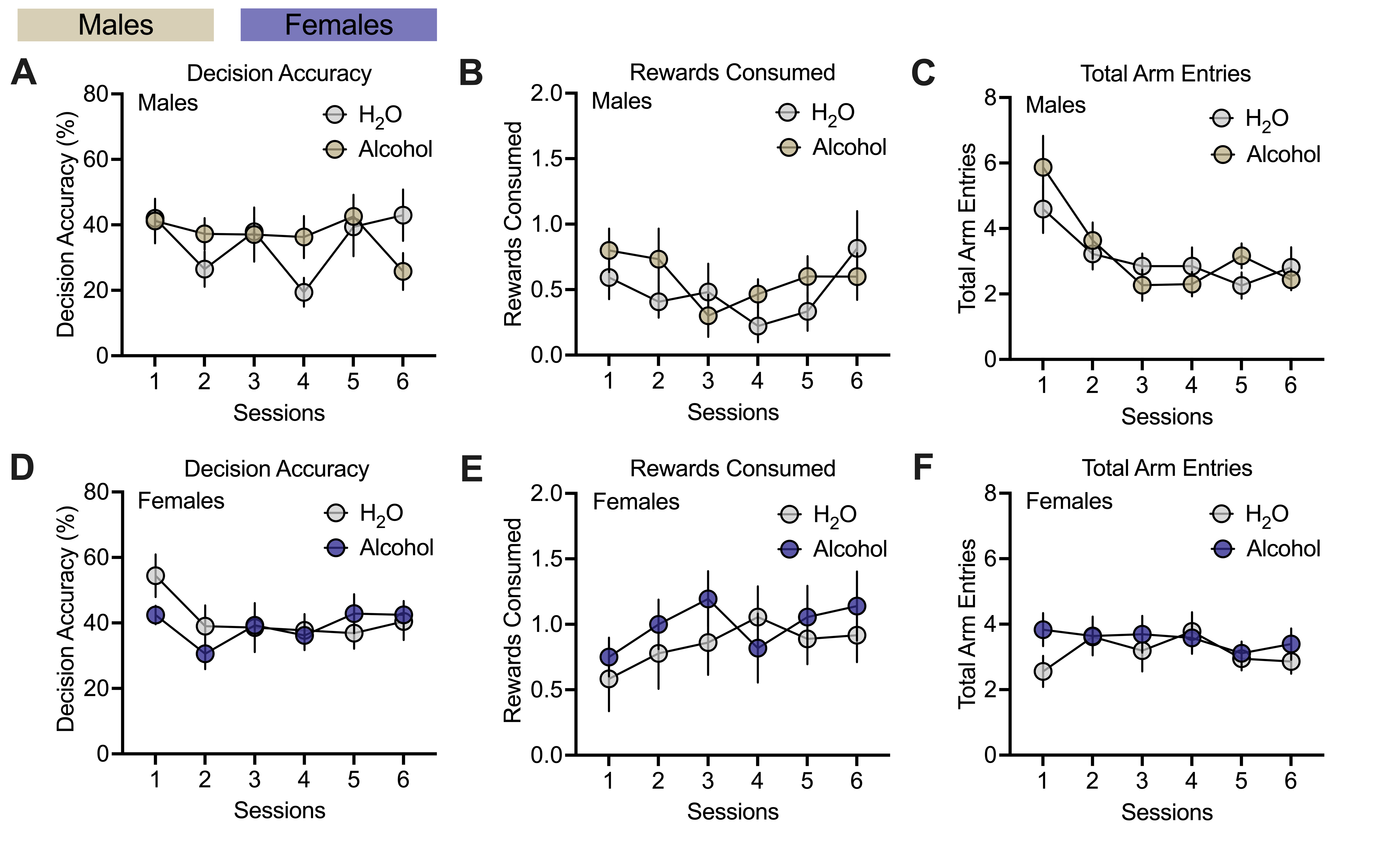
**

**Figure S1. Effects of chronic alcohol drinking on other outcomes from the radial arm maze test in male and female msP rats. A, D Decision Accuracy:** males - Alcohol: F_1,17_ = 0.21, p = 0.647, Session: F_3.73, 63.5_ = 1.49, p = 0.218, Alcohol × Session: F_5,85_ = 1.76, p = 0.129 and females - Alcohol: F_1,22_ = 0.34, p = 0.564, Session: F_4.14, 91.21_ = 1.74, p = 0.144; Alcohol × Session: F_5,110_ = 0.89, p = 0.485. **B, E Rewards Consumed**: Males - Alcohol: F_1,17_ = 0.56, p = 0.461, Session: F_3.72, 63.28_ = 1.83, p = 0.137; Alcohol × Session: F_5,85_ = 1.12, p = 0.355 and females - Alcohol: F_1,22_ = 0.31, p = 0.580, Session: F_3.44, 75.12_ = 2.28, p = 0.077, Alcohol × Session: F_5,109_ = 0.901, p = 0.483. **C, F Total Arm Entries: Males -** Alcohol: F_1,17_ = 0.13, p = 0.717, Session: F_2.81, 47.77_ = 10.07, p < 0.0001, Alcohol × Session: F_5,85_ = 1.46, p = 0.211 and females - Alcohol: F_1,22_ = 0.57, p = 0.455, Session: F_3.53, 77.12_ = 1.04, p = 0.384, Alcohol × Session: F_5,109_ = 1.10, p = 0.362.

**Table S1**. Statistical analysis of main text figures

**Abbreviations:** Prelimbic (PL), Infralimbic (IL), and hippocampus CA1 (CA1)

| **Figure** | **Dependent Variable** | **Statistical Model** | **Statistical Values** |
| --- | --- | --- | --- |
| 1C & 1D | Alcohol Intake across Time | ANOVA | *Session: F₁₆,₁₁₂ = 5.09, p < 0.001  *Sex: F₁,₇ = 19.04, p = 0.003  **Session × Sex:** F₁₆,₁₁₂ = 1.29, p = 0.2165 |
| 2B | Male Working Memory Errors | ANOVA | Alcohol: F_1,17_ = 0.08, p = 0.778  *Session: F_1.990, 33.83_ = 10.39, p = 0.0003  Alcohol × Session: F_5,85_ = 1.02, p = 0.411 |
| 2C | Females Working Memory Errors | ANOVA | Alcohol: F_1,22_ = 0.44, p = 0.510  *Session: F_3.906, 85.92_ = 3.34, p = 0.012  Alcohol × Session: F_5,110_ = 0.47, p = 0.795 |
| 2D | Male Reference Memory Errors | ANOVA | Alcohol: F_1,17_ = 3.52, p = 0.998  *Session: F_3.275, 55.68_ = 6.20, p = 0.0008  Alcohol × Session: F_5,85_ = 1.35, p = 0.251 |
| 2E | Female Reference Memory Errors | ANOVA | Alcohol: F_1,22_ = 0.97, p = 0.335  Session: F_3.789, 83.36_ = 2.13, p = 0.087  Alcohol × Session: F_5,110_ = 1.39, p = 0.233 |
| 2F | Male Reward Arm Entries | ANOVA | Alcohol: F_1,17_ = 0.44, p = 0.512  *Session: F_3.335, 56.69_ = 9.00, p < 0.0001  Alcohol × Session: F_5,85_ = 1.55, p = 0.181 |
| 2G | Female Reward Arm Entries | ANOVA | Alcohol: F_1,22_ = 0.11, p = 0.740  Session: F_3.840, 84.49_ = 0.21, p = 0.925  Alcohol × Session: F_5,110_ = 0.91, p = 0.473 |
| 3B & C | Male and Female Discrimination Index | *t*-test or  Mann-Whitney U test | *Males: U = 17.5, p = 0.029  Female: t_22_ = 0.78, p = 0.22 |
| 4A | Male and Female **PL** Insulin (*Ins*) | *t*-test | Males: t_16_ = 0.09, p = 0.464  Female: t_16_ = 1.16, p = 0.130 |
| 4B | Male and Female **IL** Insulin (*Ins*) | *t*-test | Males: t_17_ = 0.51, p = 0.307  *Female: t_14_ = 2.81, p = 0.0069 |
| 4C | Male and Female **CA1** Insulin (*Ins*) | *t-*test | Males: t_17_ = 0.10, p = 0.458  Female: t_21_= 0.66, p = 0.257 |
| 4D | Male and Female **PL** Insulin receptor (*Insr*) | *t*-test or Mann-Whitney U test | Males: U = 26, p = 0.1458  *Females: t_19_ = 2.86, p = 0.005 |
| 4E | Male and Female **IL** (Insulin receptor) *Insr* | *t*-test | Males: t_17_ = 1.02, p = 0.161  Females: t_18_ = 0.24, p = 0.389 |
| 4F | Male and Female **CA1** (Insulin receptor) *Insr* | *t*-test | Males: t_17_ = 0.447, p = 0.660  Females: t_22_= 1.54 p = 0.136 |
| 5A | Male and Female **PL** Insulin growth factor 1 (*Igf1*) | *t*-test | Males: *t*_17_ = 0.92, p = 0.185  Females: t_19_ = 1.22, p = 0.118 |
| 5B | Male and Female **IL** Insulin growth factor 1 (*Igf1*) | *t*-test | Males: t_17_ = 0.04, p = 0.483  Females: t_18_ = 0.62, p = 0.269 |
| 5C | Male and Female **CA1** Insulin growth factor 1 (*Igf1*) | *t*-test | *Males: t_17_ = 2.07, p = 0.026  Females: t_21_= 0.192, p = 0.424 |
| 5D | Male and Female **PL** Insulin growth factor 1 receptor (*Igf1r*) | *t*-test | Males: t_16_ = 1.49, p = 0.077  Female: t_19_ = 0.99, p = 0.165 |
| 5E | Male and Female **IL** Insulin growth factor 1 receptor (*Igf1r*) | *t*-test or Mann-Whitney U test | *Males: U = 16, p = 0.021  Females: t_17_ = 0.742, p = 0.234 |
| 5F | Male and Female **CA1** Insulin growth factor 1 receptor (*Igf1r*) | *t*-test or Mann-Whitney U test | Males: t_17_ = 1.57, p = 0.067  *Females: U= 33, p = 0.012 |
| 6A | Male and Female **PL** Insulin receptor substrate 1 (*Irs1*) | *t*-test | Males: t_17_ = 1.10, p = 0.1417  Females: t_17_ = 0.724, p = 0.239 |
| 6B | Male and Female **IL** Insulin receptor substrate 1 (*Irs1*) | *t*-test or Mann-Whitney U test | Males: t_17_ = 1.64, p = 0.059  Females: U = 28, p = 0.268 |
| 6C | Male and Female **CA1** Insulin receptor substrate 1 (*Irs1*) | *t*-test | *Males: t_17_ = 2.64, p = 0.008  Females: t_21_= 0.635, p = 0.266 |
| 6D | Male and Female **PL** Insulin receptor substrate 2 (*Irs2*) | *t*-test | *Males: t_15_ = 2.14, p = 0.024  *Females: t_20_ = 4.50, p = 0.0001 |
| 6E | Male and Female **IL** Insulin receptor substrate 2 (*Irs2*) | *t*-test | *Males: t_17_ = 1.84, p = 0.041  *Females: t_19_ = 4.71, p = 0.0001 |
| 6F | Male and Female **CA1** Insulin receptor substrate 2 (*Irs2*) | *t*-test | *Males: t_17_ = 2.98, p = 0.004  *Females: t_22_= 2.18, p = 0.019 |
| 7A | Male and Female **PL** Brain derived neurotrophic factor (*Bdnf*) | *t*-test | *Males: t_14_ = 1.77, p = 0.048 Females: t_19_ = 1.34, p = 0.096 |
| 7B | Male and Female **IL** Brain derived neurotrophic factor (*Bdnf*) | *t*-test or Mann-Whitney U test | Males: U = 38, p = 0.328  *Females: t_19_ = 2.31, p = 0.016 |
| 7C | Male and Female **CA1** Brain derived neurotrophic factor (*Bdnf*) | Mann-Whitney U test | Males: U = 25, p = 0.064  Females: U= 39, p = 0.051 |
| 7D | Male and Female **PL** Tyrosine kinase B (*TrkB*) | *t*-test or Mann-Whitney U test | *Males: U = 2, p = 0.0002  Female: t_20_ = 0.94, p = 0.178 |
| 7E | Male and Female I**L** Tyrosine kinase B (*TrkB*) | *t*-test | Males: t_17_ = 0.64, p = 0.264  *Females: t_20_ = 1.87, p = 0.037 |
| 7F | Male and Female **CA1** Tyrosine kinase B (*TrkB*) | *t*-test or Mann-Whitney U test | *Males: U = 23, p = 0.043  *Females: t_21_= 1.32, p = 0.09 |
| 8A | Male and Female **PL** Protein kinase m ζ (***Pkmζ*)** | *t*-test | *Males: t_17_ = 2.15, p = 0.022  Female: t_16_= 1.68, p = 0.056 |
| 8B | Male and Female **IL** Protein kinase m ζ (***Pkmζ*)** | *t*-test or Mann-Whitney U test | *Males: t_16_ = 3.51, p = 0.001  *Females: U = 18, p = 0.007 |
| 8C | Male and Female **CA1** Protein kinase m ζ (***Pkmζ*)** | *t*-test or Mann-Whitney U test | Males: t_17_ = 0.23, p = 0.409  Females: U = 42, p = 0.121 |
| 8D | Male and Female **PL** Post-synaptic density 95 (***Psd95*)** | *t*-test | Males: t_17_ = 1.47, p = 0.079  Females: t_20_ = 0.26, p = 0.396 |
| 8E | Male and Female I**L** Post-synaptic density 95 (***Psd95*)** | *t*-test or Mann-Whitney U test | Males: U = 22.5, p = 0.127  *Females: t_17_ = 2.80, p = 0.006 |
| 8F | Male and Female **CA1** Postsynaptic density 95 (***Psd95*)** | *t*-test | *Males: t_16_ = 2.24, p = 0.012  Females: t_21_= 1.181, p = 0.125**.** |

| **Table S2.** Mixed model ANOVA for radial arm maze (RAM) and novel object recognition (NOR) tests | | | |
| --- | --- | --- | --- |
| **Figure** | **Dependent Variable** | **Statistical Model** | **Statistical Values** |
| 2B & 2C | Working Memory Errors | 3-way Repeated Measures ANOVA | Sex: F_1,39_ = 0.80, p = 0.376  Alcohol: F_1,39_ = 0.08, p = 0.778  *Session: F_5,195_ = 11.05, p < 0.001  **Session × Sex× Alcohol:** F_5,195_ = 0.86, p = 0.507 |
| 2D & 2E | Reference Memory Errors | 3-way Repeated Measures ANOVA | Sex: F_1,39_ = 0.16, p = 0.687  Alcohol: F_1,39_ = 0.42, p = 0.518  *Session: F_5,195_ = 5.03, p < 0.001  **Session × Sex× Alcohol:** F_5,195_ = 0.625, p = 0.681 |
| 2F & 2G | Reward Arm Entries | 3-way Repeated Measures ANOVA | Sex: F_1,39_ = 1.15, p = 0.290  Alcohol: F_1,39_ = 0.41, p = 0.525  *Session: F_5,195_ = 6.63, p < 0.001  **Session × Sex× Alcohol:** F_5,195_ = 1.90, p = 0.095 |
| S1A & S1D | Decision Accuracy | 3-way Repeated Measures ANOVA | Sex: F_1,39_ = 2.37, p = 0.132  Alcohol: F_1,39_ = 0.001, p = 0.973  *Session: F_5_*_,_*_195_ = 2.76, p < 0.019  **Session × Sex× Alcohol:** F_5,195_ = 1.76, p = 0.122 |
| S1B & S1E | Rewards Consumed | 3-way Repeated Measures ANOVA | *Sex: F_1,38_ = 4.65, p = 0.037  Alcohol: F_1,38_ = 0.35, p = 0.556  Session: F_5_*_,_*_190_ = 0.83, p = 0.526  **Session × Sex× Alcohol:** F_5,190_ = 1.52, p = 0.185 |
| S1C & S1F | Total Arm Entries | 3-way Repeated Measures ANOVA | Sex: F_1,38_ = 0.03, p = 0.849  Alcohol: F_1,38_ = 0.27, p = 0.605  *Session: F_5_*_,_*_190_ = 6.10, p < 0.001  **Session × Sex × Alcohol:** F_5,190_ = 0.839, p = 0.523 |
| 3B & C | Discrimination index | 2-way ANOVA | Sex: F_1,38_ = 1.31, p = 0.258  *Alcohol: F_1,38_ = 4.65, p = 0.037  Sex ***×* Alcohol:**  F_1,38_ = 1.442, p = 0.237 |
